# Iatrogenic Cushing’s syndrome with inhaled steroid plus antidepressant drugs

**DOI:** 10.1186/2049-6958-7-26

**Published:** 2012-08-29

**Authors:** Ozlem Celik, Mutlu Niyazoglu, Hikmet Soylu, Pinar Kadioglu

**Affiliations:** 1 Division of Endocrinology and Metabolism, Department of Internal Medicine, Cerrahpasa Medical School, University of Istanbul, Istanbul, Turkey

**Keywords:** Cushing’s syndrome, Fluticasone propionate, Mirtazepine, Paroxetine

## Abstract

Current guidelines recommend the use of inhaled corticosteroids (ICS) for suppression of airway inflammation in patients with asthma. Although it is well known that ICS cause dose-related adrenocortical suppression, it is less known that they can lead to iatrogenic Cushing’s syndrome (CS). Fluticasone propionate (FP) is an ICS more potent than beclomethasone and budesonide. FP is metabolized as mediated by cytochrome P450 3A4 in the liver and the gut. Systemic bioactivity of FP can increase with the use of drugs that affect the cytochrome P450. Herein, we report the rapid development of iatrogenic CS in a patient receiving paroxetine and mirtazepine for 12 weeks in addition to inhaled FP.

## Background

Inhaled corticosteroids (ICS) are widely accepted as the first line of treatment for the suppression of airway inflammation of asthma [1,2]. Although it is well known that ICS cause dose-related adrenocortical suppression, it is less known that they can lead to iatrogenic Cushing’s syndrome (CS) [3-5]. Fluticasone propionate (FP) is the most potent inhaled corticosteroid and is highly lipophilic with a large volume of distribution. Low plasma concentrations of FP are achieved after inhaled dosing due to first-pass metabolism and high systemic clearance, mediated by cytochrome P450 3A4 in the liver and the gut [6]. Systemic bioactivity of FP depends on: glucocorticoid potency, dose, duration of therapy, individual glucocoticoid receptor sensitivity, and drug combinations. Previously, iatrogenic CS was recognized after prolonged high dose use of FP in a female patient [3]. The literature presents many reports of iatrogenic CS with osteoporosis and secondary adrenal failure in Human Immunodeficiency Virus (HIV)-infected patients receiving ICS and ritonavir [7].

Paroxetine and mirtazepine are antidepressant drugs that inhibit cytochrome P450 and, consequently, decrease the clearance of corticosteroids. Paroxetine is highly metabolized with cytochrome P450- dependent CYP2D6. There is a moderate effect on other P450 enzymes. Although mirtazepine is a weak competitive inhibitor of CYP 1A2, 2D6 and 3A4, it is known that there have been no clinical effects on cytochrome P450 to date [8].

Herein, we report for the first time the rapid development of iatrogenic CS in a patient who has been taking paroxetine and mirtazepine, in addition to inhaled FP, for 12 weeks.

## Case presentation

**Figure 1:**
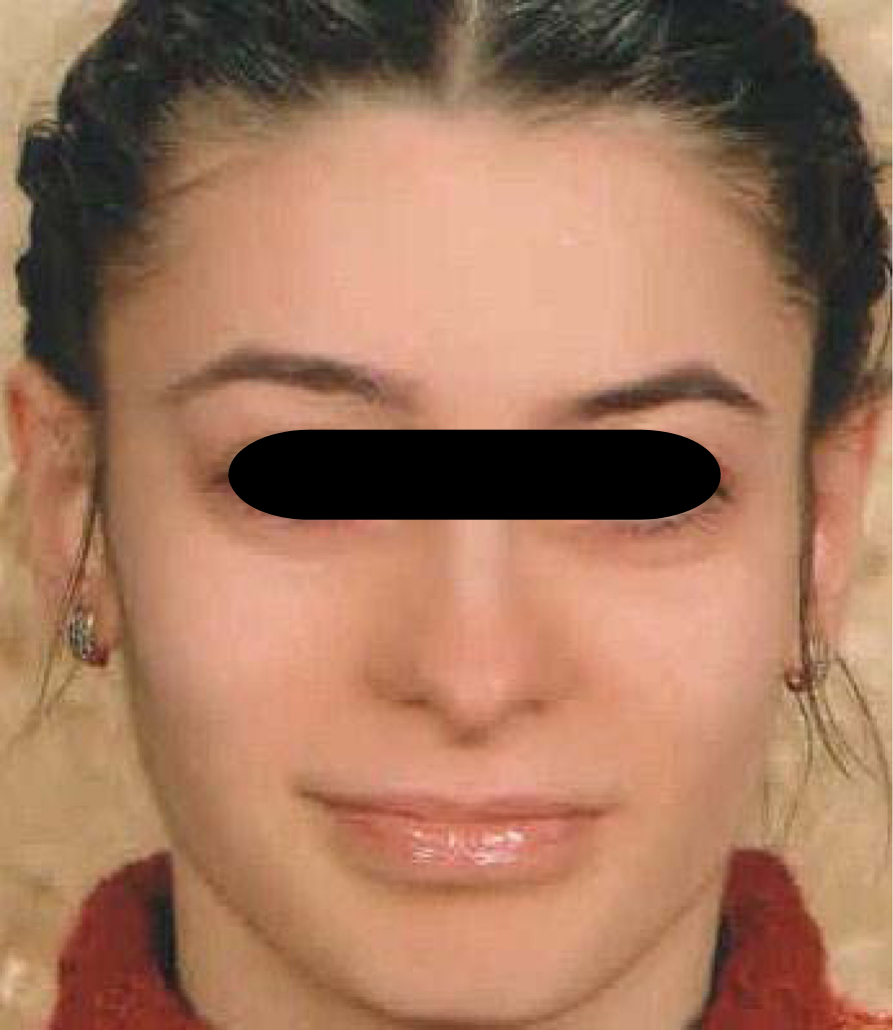
Patient’s facial appearance before starting inhaled fluticasone propionate and antidepressants.

**Figure 2:**
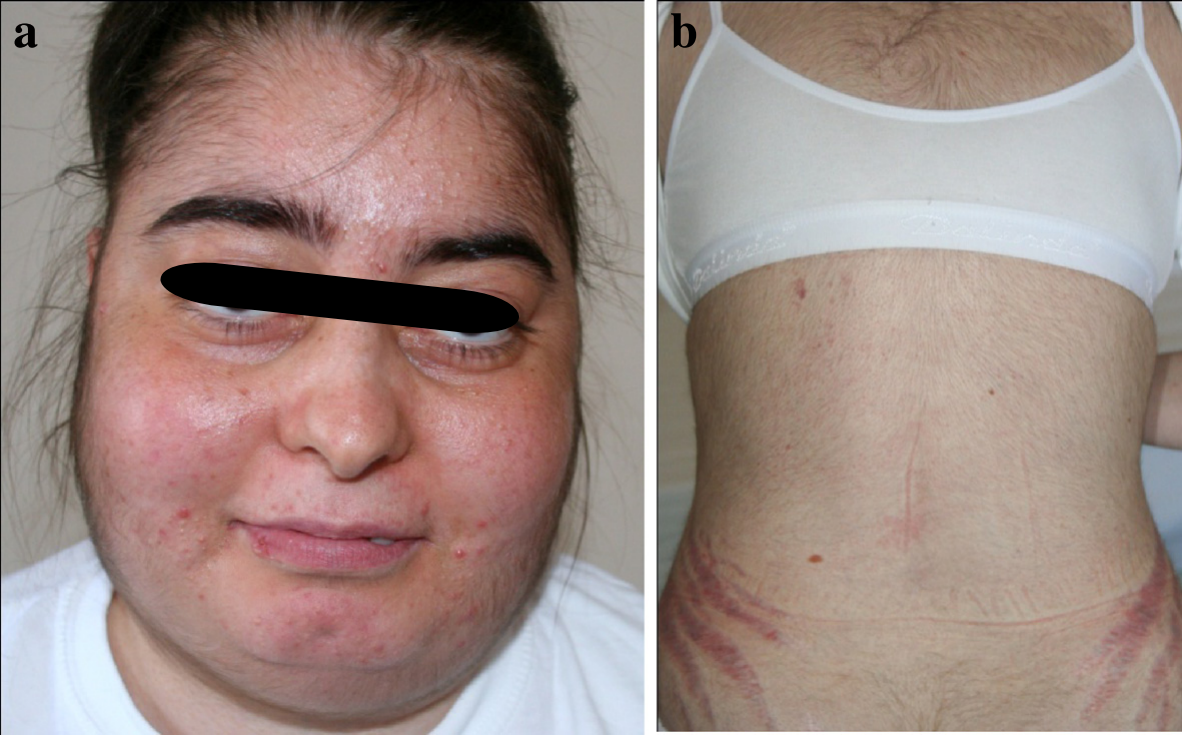
**a - Patient’s facial appearance 3 months after therapy with inhaled fluticasone propionate and antidepressants**. **b - Hirsutism and strias were shown in the patient with iatrogenic Cushing’s syndrome.**

A 30-year-old female patient who had weakness, hirsutism, acne, increased abdominal fat and purple strias was admitted to the Endocrinology and Metabolism out-patient clinic of Cerrahpasa Medical School, University of Istanbul. She had been diagnosed with depression and asthma three months earlier. The patient had been receiving inhaled FP (500 mcg twice daily) and paroxetine (20mg/day, *po*.), mirtazepine (30 mg/day, *po.*) therapies with no evidence of CS (Figure 1). The patient complained of central weight gain, muscle wasting, weakness, easy bruising, hirsutism, acne and abdominal striae after treatment with FP for three months. Obvious moon face (Figure 2a), facial plethora, acne, oral mucositis, increased truncal and abdominal fat, buffalo hump, proximal myopathy and hirsutism were noted at the physical examination. Prominent striae were evident on her abdomen and arms (Figure 2b). Blood pressure was 135/80 mmHg with no postural drop. Sub-acute thrombosis, caused by edema and swelling in the right leg, was detected by Doppler USG in the patient’s right popliteal vein. Enoxaparin sodium 0.6 ml twice daily was started. The patient was not osteoporotic.

An early morning cortisol of 0.8 μg/dl, ACTH of 1 pg/ml and a urinary free cortisol excretion of 60 mg/day were determined. Initial laboratory results are shown in Table 1. A short synacthen test (1 μg) was clearly abnormal, rising to 3μg/dl and 3.4 μg/dl at 30 and 60 minutes, respectively.

**Table 1:** **The initial laboratory results in patient with Iatrogenic Cushing’s syndrome**

	**Results (normal range)**		**Results (normal range)**
ACTH	: **1 pg/mL** (0–46)	Albumin	: 3.7 gr/dL (3.2-4.8)
Cortisol	: **0.8 μg/dL** (5–25)	AST	: 41U/L (0–45)
Urinary free cortisol	: 60 μg /day (20–70)	ALT	: 21U/L(10–45)
DHEAS	: **9 μg/dL** (35–430)	Alkaline phosphatase	: 42U/L (45–129)
Total testosterone	:** 15ng/dL** (20–90)	Calcium	: 9 mg/dL (8.6-10)
Free T4	: 2.34ng/dl (0.8-1.58)	Phosphorus	: 2.9mg/dL (2.1-4)
TSH	: 1.44mIU/L (0.4-4)	Total cholesterol	: 232mg/dL (50–200)
Glucose	: **104mg/dL** (74–106)	Triglyceride	: 68mg/dL(20–170)
Urea	: 21mg/dL (19–50)	HDL cholesterol	: 79mg/dL(45–65)
Creatinine	: 0.6mg/dL (0.7-1.3)	LDL cholesterol	: 139 mg/dL(5–130)
Sodium	: 143mEq/L (135–145)	CRP	: < 3mg/L (0–5)
Potassium	: **3.2 mEq/L** (3.5-5.5)	ESR	: 9 mm/sa (5–25)
Leukocyte	: 7700/mm^3^ (4600–10.000)	Hemoglobin	: 13 gr/dL(12–17)
Hematocrit	: 38% (37–50)	Platelet	: **133000/mm**^**3**^(150–450)
FEV_1_	: 2.67 L (2.83)	FEV_1_/ FVC	: 97% (83)
PEF	: 6.16L/sec (6.59)	MEF25-75	: 4.51L/sec (3.83)

Cortisol after 1 μg ACTH stimulation test (30^th^ and 60^th^): **3 and 3.4 μg/dl** (> 18 μg/dl).

Definition of abbreviations: ALT, serum alanine amino-transferase; AST serum amino-transferase; CRP, C reactive protein; DHEAS, dehydroepiandrosterone sulfate; ESR, erythrocyte sedimentation rate; FEV, forced expiratory volume; FVC, forced vital capacity; MEF 25–75, mean forced expiratory flow between 25% and 75% of FVC; PEF, peak expiratory flow.

After consultation with a thoracic diseases specialist, the patient was reassessed for asthma. Respiratory function tests were as follows: FEV_1_: 2.67, FEV_1_/FVC: 97, PEF: 6.16, MEF_25-75_: 4.51. FP was changed to salbutamol sulphate as needed for a re-evaluation of the diagnosis.

The patient developed symptoms of extreme fatigue, anorexia, and myalgia after cessation of FP. Hydrocortisone (20 mg /day for 2 months, *po*), at gradually decreasing doses, was started for evaluation of the hypothalamic-pituitary-adrenal axis. Two months after withdrawal of FP, the patient felt that her clinical appearance was better, but there was still evidence of hypothalamic-pituitary-adrenal (HPA) axis suppression.

## Discussion

A case of clinical CS secondary to increased systemic concentration of FP due to cytochrome P450 inhibition has been described. The condition of the patient was associated with subsequent adrenal insufficiency related to the suppression of pituitary-adrenal axis.

FP undergoes very high first-pass hepatic metabolism, mediated by cytochrome P450 3A4, and it is also very lipophilic, resulting in much higher tissue levels, longer plasma half-life and greater glucocorticoid receptor affinity than other ICSs such as beclomethasone and budenoside [9]. Recently, HPA axis suppression related to ICS is thought to be due to systemic absorption through the lungs rather than oral absorption via ingested corticosteroids after inhalation [10]. A recent retrospective study [11] aimed at estimating the incidence of adrenal insufficiency in users of inhaled corticosteroids reported 46 cases (involving fluticasone, budesonide, or beclomethasone). Eleven patients treated with fluticasone and four with budesonide were concomitantly treated with an enzymatic inhibitor: itraconazole (six cases), ritonavir (five cases), verapamil (two cases), and diltiazem (two cases). Although the use of ICS in the long-term management of asthma is recommended, it is advised that the lowest effective dose of ICS be used to prevent systemic side effects [1,12].

A recent meta-analysis showed that FP had minimal effects on adrenal function when prescribed within the therapeutic range of 50–500 μg/day to patients with asthma [13]. It was demonstrated that age, being a normal volunteer inhaler technique and compliance, plays a role in determining the systemic effects of ICS. In spite of this, Paton J et al. reported that clinical adrenal insufficiency was particularly associated with high doses (> 400 μg/day) of inhaled FP in children with asthma [4]. Wilson AM et al. demonstrated that high doses of inhaled FP alone were associated with adrenal suppression and CS features with use over 2 years in a female asthmatic patient. Inhaled FP was changed to budenoside, then her cushingoid features improved and HPA also returned to normal [3]. Recently Matos AC et al. presented a 22- year-old man with adrenal insufficiency and a cushingoid habitus who was receiving 250 μg FP and salmeterol 50 μg two blisters, twice a day for four years [10]. These cases confirms that HPA axis suppression with features of Cushing’s syndrome occurs at commonly used, and licensed, doses of ICS.

There is more evidence in the literature of iatrogenic CS associated with the co-administration of inhaled FP with both high doses and low doses of ritonavir used in the treatment of HIV infection. Samaras K et al. reported six patients with iatrogenic CS who had HIV infection and were receiving ritonavir and FP combination. These patients demonstrated significant disturbance of mood, osteoporosis and adrenal insufficiency requiring supportive treatment after FP withdrawal [7]. Clinical CS was shown in a patient with asthma receiving itraconazol and FP combination due to reduction in FP clearance by inhibition of cytochrome P450 3A4 [14]. Hoover WC et al. reported a 9- year-old girl with cystic fibrosis (CF)-related liver disease and asthma. Seven weeks after the initiation of inhaled fluticasone, she developed vaginal candidiasis and was prescribed fluconazole 100 mg/day. Iatrogenic Cushing syndrome was shown in this patient with inhaled FP and a CYP3A4 inhibitor, fluconazole [15]. Table 2 shows that several drugs inhibit cytochrome P450 dependent CYP3A4 and CYP2D6 and consequently decrease the clearance of synthetic glucocorticoids. Importantly, adrenal suppression does not occur in every patient treated with a combination of these drugs and ICS. Variation in CYP3A4 activity, in glucocorticoid receptor sensitivity, or in glucocorticoid receptor polymorphism, in addition to patient age, may influence the response of the individual. There are reported cases of children and adults with cushingoid features and adrenal suppression due to budesonide with CYP3A4 inhibitors [16].

**Table 2:** Some important drugs that inhibit cytochrome P450 dependent CYP3A4 and CYP2D6

**CYP2D6**	**CYP3A4**
Antiarrhythmics (Flecainide, Propafenone, etc.)	Antiarrhythmics (Amiodarone, Lidocaine, Propafenone, etc.)
Beta blockers (Carvedilol, Metoprolol, etc..)	Anti-histamines (Astemizole, Chlorpheniramine)
Neuroleptics (Haloperidol, Risperidone, Clozapine, etc.)	Anti-cancer (Tamoxifen, Vinblastine)
Opiates (Dextromethorphan, Codeine, Tramadol, etc.)	Benzodiazepines (Midozalam, Diazepam, Alprazolam, etc.)
SSRI (Fluoxetine, Paroxetine, Fluvoxamine, etc.)	HIV-protease inh (Ritonavir, Indinavir, etc.)
Tricyclic antidepressants (Amitriptyline, Desipramine, Imipramine, Nortriptyline, etc.)	HMG CoA reductaseinh (Atorvastatin, Lovastatine, Simvastatin)
	Hormones (Cortisol, Progesterone, Testosterone, steroids)
	Calcium channel blocker (Diltiazem, Verapamil, Nitrendipin)
	Macrolides (Erythromycin, Clarithromycin)
	Others (Ketoconazole, Itraconazole, Lidocaine, Cocaine,…etc)

Paroxetine and mirtazepine are antidepressant drugs that inhibit cytochrome P450, and, consequently, decrease the clearance of corticosteroids. Although it is known that paroxetine is highly metabolized with cytochrome P450-dependent CYP2D6, mirtazepine has not known to have clinical effects on cytochrome P450 to date [8]. However, it should be verified with research and observations. It was thought that our patient had developed clinical CS secondary to increased systemic concentration of FP due to cytochrome P450 inhibition by antidepressants. In addition to the CS development, biochemical results of the patient showed low cortisol, ACTH, testosterone, DHEAS (Dehydroepiandrosterone sulfate) levels, and hypokalemia. Moreover, she had impaired fasting glucose. Recently, declines in DHEAS after the initiation of ICS have been recommended as an early index of adrenal suppression [17]. Significantly low DHEAS levels (9 μg/dl) were found in this patient. These findings may provide significant clinical utility for the early detection of adrenal suppression after ICS therapy.

## Conclusions

In conclusion, to the best of our knowledge, this is the first report of a patient with iatrogenic CS receiving FP and antidepressants. Both thoracic diseases specialists and general practitioners should be aware of the risk of iatrogenic CS due to serious drug interactions with FP.

## Consent

Written informed consent was obtained from the patient for publication of this case report and any accompanying images. A copy of the written consent is available for review by the Editor-in-Chief of this journal.

## Competing interests

The authors declare that they have no competing interests.

## Funding

This research did not receive any specific grant from any funding agency in the public, commercial, or not-for-profit sector.
